# Detailed Postmortem Profiling of Inflammatory Mediators Expression Revealed Post-inflammatory Alternation in the Superior Temporal Gyrus of Schizophrenia

**DOI:** 10.3389/fpsyt.2021.653821

**Published:** 2021-03-18

**Authors:** Ryuta Izumi, Mizuki Hino, Akira Wada, Atsuko Nagaoka, Takashi Kawamura, Tsutomu Mori, Makoto Sainouchi, Akiyoshi Kakita, Kiyoto Kasai, Yasuto Kunii, Hirooki Yabe

**Affiliations:** ^1^Department of Neuropsychiatry, School of Medicine, Fukushima Medical University, Fukushima, Japan; ^2^Department of Psychology, Takeda General Hospital, Aizuwakamatu, Japan; ^3^Department of Neuropsychiatry, Graduate School of Medicine, The University of Tokyo, Tokyo, Japan; ^4^Department of Human Life Sciences, School of Nursing, Fukushima Medical University, Fukushima, Japan; ^5^Department of Pathology, Brain Research Institute, Niigata University, Niigata, Japan; ^6^Department of Disaster Psychiatry, International Research Institute of Disaster Science, Tohoku University, Sendai, Japan

**Keywords:** schizophrenia, postmortem, IP10, IL1A (IL1α), superior-temporal gyrus, inflammatory meadators

## Abstract

Recent studies have lent support to the possibility that inflammation is associated with the pathology of schizophrenia. In the study of measurement of inflammatory mediators, which are markers of inflammation, elevated inflammatory cytokine levels in the brain and blood have been reported in patients with schizophrenia. Several postmortem brain studies have also reported changes in the expression of inflammatory cytokines. However, it is not clear how these elevated inflammatory cytokines interact with other inflammatory mediators, and their association with the pathology of schizophrenia. We comprehensively investigated the expression of 30 inflammatory mediators in the superior temporal gyrus (STG) of 24 patients with schizophrenia and 26 controls using a multiplex method. Overall, inflammatory mediator expression in the STG was mostly unchanged. However, the expression of interleukin (IL)1-α and interferon-gamma-inducible protein (IP)-10 was decreased [IL-1α, median (IQR), 0.51 (0.37–0.70) vs. 0.87 (0.47–1.23), *p* = 0.01; IP-10, 13.99 (8.00–36.64) vs. 30.29 (10.23–134.73), *p* = 0.05], whereas that of IFN-α was increased [2.34 (1.84–4.48) vs. 1.94 (1.39–2.36), *p* = 0.04] in schizophrenia, although these alterations did not remain significant after multiple testing. Clustering based on inflammatory mediator expression pattern and analysis of upstream transcription factors using pathway analysis revealed that the suppression of IL-1α and IP-10 protein expression may be induced by regulation of a common upstream pathway. Neuroinflammation is important in understanding the biology of schizophrenia. While neuroimaging has been previously used, direct observation to determine the expression of inflammatory mediators is necessary. In this study, we identified protein changes, previously unreported, using comprehensive protein analysis in STG. These results provide insight into post-inflammatory alternation in chronic schizophrenia.

## Introduction

Schizophrenia is a psychiatric disorder with a lifetime prevalence of ~1%. While the pathogenesis of schizophrenia is yet to be fully characterized (1), an association between neuroinflammation and schizophrenia has been intimated from various studies. Studies on plasma cytokines levels have reported elevated levels of major inflammatory cytokines such as IL-1β, TNF-α, and INF-γ; low-grade systemic inflammation has been shown to be present in first-episode psychosis (FEP) and pre-onset state of schizophrenia (2, 3). Prenatal maternal infections are epidemiologically established risk factors for schizophrenia. Animal studies have demonstrated that exposure to perinatal inflammatory mediators (e.g., epidermal growth factor-EGF) induced schizophrenia-like cognitive and behavioral abnormalities after maturation (4, 5). Indirect observations such as neuroimaging indicate that neuroinflammation occurs in the brain of patients in the acute phase of schizophrenia. Positron emission tomography (PET) studies have revealed that activated microglia were increased in the frontal and temporal lobes and in the total gray matter in patients with acute-phase schizophrenia (6, 7).

Renewed interest in the relationship between schizophrenia and inflammation is occasioned by advances in genetic studies that led to the identification of associations between genes involved in the regulation of the immune system and increased risk of schizophrenia (8).

In contrast, postmortem studies may reveal post-inflammatory alternation associated with the pathology of chronic schizophrenia. Genetic predispositions and diverse prenatal maternal infections predispose the individual to the development of schizophrenia by lowering the threshold of driving the neuroinflammatory system. Moreover, repetitive neuroinflammation caused by exposure to low stimulation levels, such as stress and infection, triggers the onset of acute psychosis by disturbing neurotransmission and post-sensitization changes, which further decrease the threshold (9). Previous postmortem studies have consistently reported increased gene expression of IL1β in schizophrenia. Besides postmortem studies investigating protein expression levels in the brain, individual recent studies have reported elevated levels of inflammatory cytokines such as TNF-α and IL-6 (10, 11). However, to date, no meta-analysis has confirmed consistent changes in protein expression levels (12, 13). Most previous postmortem studies have examined inflammatory mediators in the PFC, dorsolateral prefrontal cortex (DLPFC), and hippocampus, with only few having been performed in other brain regions. In addition, these previous studies have focused on evidence of inflammatory cytokines such as IL-1β, IL-6, and TNF-α, with few comprehensive measurements involving other inflammatory mediators in the superior temporal gyrus (STG) (12, 13).

The STG is a brain region suspected to be associated with hallucinations and thought disorders in the context of the pathological mechanisms characteristic of schizophrenia. Based on studies of event-related potentials in electroencephalography, the STG has been thought to be the brain region that is the major generator of mismatch negativities reported to be abnormal in schizophrenia (14). In addition, structural and functional alterations in the STG in schizophrenia have been observed in recent imaging studies, such as fMRI studies, thereby strengthening the pathological association between the STG and schizophrenia (15).

Therefore, the purpose of the present study is to provide insight that complement previous studies by comprehensively assessing inflammatory mediators in the superior temporal gyrus of schizophrenia using multiplex immunoassays.

## Materials and Methods

### Human Postmortem Brain Tissue Collection and Characterization

Postmortem brain tissue samples from patients with schizophrenia (cases) and control subjects were obtained from Fukushima Brain Bank at the Department of Neuropsychiatry, Fukushima Medical University, and Brain Research Institute at Niigata University, as described previously (16). Use of postmortem human brain tissues was approved by the Ethics Committee of Fukushima Medical University and Niigata University, and the study complied with the Declaration of Helsinki and its later amendments. Each patient with schizophrenia fulfilled the diagnostic criteria established by the American Psychiatric Association (Diagnostic and Statistical Manual of Mental Disorders: DSM-IV). Control, on the other hand, refers to individuals who had not been diagnosed with a mental illness during lifetime according to DSM-IV. Detailed demographic information of each group is summarized in Table 1.

**Table 1:** Subject demographics and clinical characteristics.

**Variables**	**Controls**	**Schizophrenia**	***P*-value**
Number of samples	26	24	
**Sex**			
Female	11	9	0.73^b^
Male	15	15	
**Age at death^a^**			
(years)	62.15 ± 16.21	68.42 ± 11.03	0.12^c^
**PMI^a^**			
(hour)	9.45 ± 11.32	16.31 ± 11.60	0.04^c^^※^
pH^a^	6.21 ± 0.33	6.30 ± 0.41	0.39^c^
CPZ-eq^a^	-	529.54 ± 586.72	

*PMI, postmortem interval; CPZeq, chlorpromazine equivalent dose*.

a *Data are reported as Mean ± standard deviation*.

b *χ^2^-test*.

c *Student's t-test*.

※ *P < 0.05*.

### Protein Expression Analysis by Multiplex Assay

Pieces (about 100 mg) of gray matter from STG (Brodmann Area:BA22) were picked from frozen brain. The tissues were suspended in N-PER™ Neuronal Protein Extraction Reagent (Thermo Fisher Scientific, USA) and sonicated using a sonicator (W385, Heatsystems, USA). The samples were diluted twice with phosphate-buffered saline (PBS; 137 mM NaCl, 2.7 mM KCl, 10 mM Na2HPO4, 1.76 mM KH2PO4). The samples were centrifuged at 12,000 × g for 5 min and filtered (Ultrafree-MC-GV, 0.22 μm, Merck Milipore, Bedford, MA, USA). The protein concentration was determined by the Bradford method (Bradford protein assay kit,Bio-Rad Laboratories, Hercules, CA) with bovine serum albumin (BSA) as the standard. The final concentration of total protein of the samples for the multiplex analysis was around 4 mg/ml.

The levels of IL-1β, GM-CSF, IFN-γ, IL-2, IL-4, IL-5, IL-6, IL-7, IL-8, IL-10, IL-12 (p40), IL-13, MCP-1, TNF-α, Eotaxin, G-CSF, IFN-α2, IL-1α, IL-3, IL-12 (p70), IL-15, IL-17, IP-10 (CXCL-10), MIP-1α, MIP-1β, TNF-β, EGF, IL-1RA, MDC (CCL22), and VEGF in the samples were determined by multiplex fluorescent bead based immunoassays (hereinafter referred to as the “multiplex assay”). The kits (MAPmateTM, Merck Millipore, Tokyo, Japan) used in this study were Milliplex MAP Human Cytokine/Chemokine Panel 1 Pre-mixed 29Plex (Cat.# F-MIL-HCYTMAG-60K-PX29). The level of each protein was normalized against the total protein concentration.

### Statistical Analysis

Demographic variables (sex, age, pH and PMI; postmortem interval) were compared between groups using a chi square-test and Student's *t*-test, for categorical and continuous variables, respectively. Data of protein expression were compared between groups using a Mann-Whitney *U*-test. Thereafter, a Benjamini-Hochberg procedure was conducted for multiple testing.

A Spearman's rank test was used for correlation analysis between protein expression levels, and confounding factors including age, sex, chlorpromazine equivalents (CPZeq), pH and PMI. *P* < 0.05 was considered statistically significant. SPSS ver. 25.0 (SPSS, Chicago, IL, USA) was used for the analysis.

In order to clarify the relationship between each inflammatory mediator, we tried clustering by expression pattern. A hierarchical cluster analysis using the Ward method based on the Euclidean squared distances was performed to evaluate the expression patterns of the 20 inflammatory mediators, for which the expression levels could be measured in all cases, including the schizophrenia group and the control group. At this time, all measurements were standardized using a continuous variable z-score. This hierarchical cluster analysis generated a dendrogram and classified inflammatory mediators into three clusters at the 20 rescaled distance cluster combine.

In addition, an upstream transcription factor analysis was performed to evaluate the validity of clustering and to connect with neuroinflammation signaling cascades. QIAGEN's Ingenuity® Pathway Analysis (IPA®, QIAGEN Redwood City, www.qiagen.com/ingenuity) was applied to identify common upstream transcription factors of inflammatory mediators belonging to each cluster. The 10 transcription factors most relevant to the inflammatory mediator within each cluster were assessed, based on expected effects between transcriptional regulators and their target genes from published literature citations that have been curated and stored in the IPA program (17).

## Results

### Comparison of Protein Expressions Between Case and Controls

**Figure 1:**
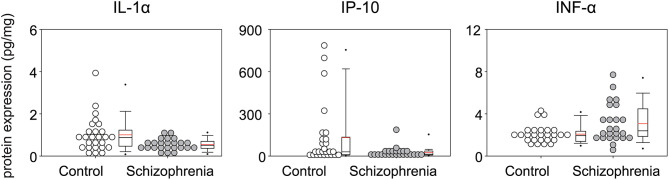
Multiplex quantification of proteins in the superior temporal gyrus (Brodmann area; BA 22) of patients with schizophrenia and controls. Cytokine concentration (pg) of Interleukin (IL-1α), Interferon-gamma-inducible protein 10 (IP-10), Interferon α (IFN α) in 1 mg of total protein.

The results of analysis of the inflammatory mediators expression levels in STG are shown in Table 2. Of the 30 proteins analyzed, 10 proteins [eotaxin, IFN-γ, IL-17, IL-2, IL-3, IL-4, IL-5, TNF-β, MDC (CCL22), IL-12 (p70)] could not be quantified. The median (IQR) of IL-1α and IP-10 levels were lower in patients with schizophrenia (IL-1α [pg/mg], 0.51 (0.37–0.70) vs. 0.87 (0.47–1.23), *p* = 0.01; and IP-10 [pg/mg], 13.99 (8.00–36.64) vs. 30.29 (10.23–134.73), *p* = 0.05], while the median (IQR) IFN-α [pg/mg] level was higher [2.34 (1.84–4.48) vs. 1.94 (1.39–2.36), *p* = 0.04], although these alterations did not remain significant after multiple testing (Figure 1).

**Table 2:** The results of the analysis of expression levels of 20 cytokines in the superior temporal gyrus (STG).

**Cytokines (pg/mg)^a^**	**Controls**	**Schizophrenia**	**Mann-Whitney *U*-test**	**Benjamini-Hochberg procedure**
	**Median (p25–p75)^b^**	**Median (p25–p75)^b^**	***P*-value**	***P*-value**
EGF	0.54 (0.30–0.73)	0.54 (0.43–1.01)	0.62	0.93
G-CSF	1.66 (1.04–6.60)	3.00 (1.41–5.28)	0.36	0.93
GM-CSF	0.15 (0.01–0.45)	0.02 (0.00–0.25)	0.10	0.50
INF-α	1.94 (1.39–2.36)	2.34 (1.86–4.48)	0.04^*^	0.33
IL-10	0.24 (0.07–0.49)	0.15 (0.08–0.27)	0.52	0.93
IL-12P40	0.43 (0.03–0.96)	0.16 (0.00–0.82)	0.44	0.93
IL-13	0.81 (0.55–1.06)	0.72 (0.51–1.17)	0.97	0.97
IL-15	2.35 (1.69–3.84)	2.23 (1.58–3.47)	0.56	0.93
IL-1RA	24.23 (17.68–30.00)	17.50 (14.95–25.39)	0.21	0.84
IL-1α	0.87 (0.47–1.23)	0.51 (0.37–0.70)	0.01^*^	0.2
IL-1β	0.05 (0.01–0.14)	0.05 (0.00–0.11)	0.97	0.97
IL-6	1.61 (0.24–11.34)	0.87 (0.23–7.09)	0.90	0.95
IL-7	0.09 (0.00–0.32)	0.08 (0.00–0.30)	0.73	0.95
IL-8	5.53 (1.99–23.80)	3.10 (2.01–6.79)	0.33	0.93
IP-10	30.29 (10.23–134.73)	13.99 (8.00–36.64)	0.05^*^	0.33
MCP-1	37.17 (22.55–93.87)	34.63 (17.58–87.75)	0.62	0.93
MIP-1α	0.26 (0.04–1.14)	0.33 (0.00–2.15)	0.86	0.95
MIP-1β	1.35 (0.29–3.81)	1.18 (0.48–2.17)	0.76	0.95
TNF-α	0.23 (0.05–0.99)	0.19 (0.00–0.38)	0.43	0.93
VEGF	0.91 (0.00–2.27)	0.75 (0.19–2.10)	0.65	0.93

a *Cytokine concentration (pg) in 1 mg of the total protein*.

b *Data are reported as median [25th percentile (p25)-75th percentile (p75)]*.

* * P < 0.05*.

### Correlation Between Protein Expression and Patient Clinical Characteristics

The results of the correlation between each clinical factor in patients with schizophrenia and the expressions of IL-1α, IP-10, and IFN-α in STG are shown in Table 3. Expression of IP-10 was negatively correlated with PMI, and Expression of IFN-α was positive correlated with pH. No significant correlation was observed between other factors and protein expressions.

**Table 3:** Correlation of the expression levels of cytokines with confounding factors analyzed in the Spearman's rank test.

**Cytokine**	**Confounding factors**			
	**Age**	**PMI**	**pH**	**CPZeq**
INF-α	*r* = −0.22 (*p* = 0.88)	*r* = 0.25 (*p* = 0.08)	*r* = 0.33 (*p* = 0.02)^*^	*r* = 0.27 (*p* = 0.20)
IL-1α	*r* = 0.12 (*p* = 0.39)	*r* = −0.18 (*p* = 0.22)	*r* = −0.01 (*p* = 0.92)	*r* = −0.03 (*p* = 0.88)
IP-10	*r* = −0.08 (*p* = 0.60)	*r* = −0.34 (*p* = 0.02)^*^	*r* = −0.09 (*p* = 0.56)	*r* = 0.39 (*p* = 0.06)

*PMI, postmortem interval; CPZ eq, chlorpromazine equivalent dose*.

* *P < 0.05*.

### Cluster Analysis of the Expression Patterns of Inflammatory Mediators

**Figure 2:**
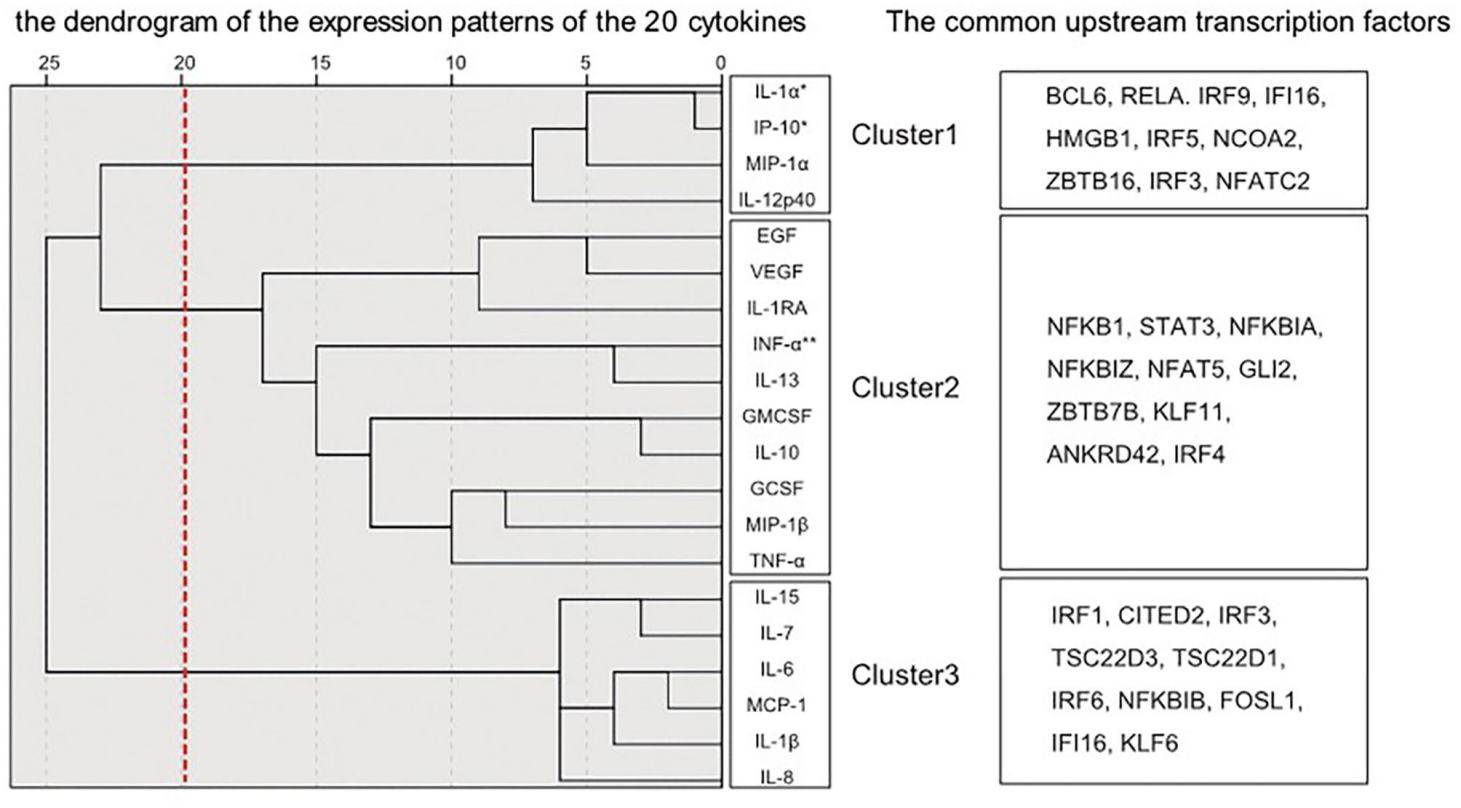
Dendrograms generated by hierarchical cluster classification of cytokine expression patterns. The hierarchical cluster analysis classified cytokines into three clusters. The transcription factors upstream of each of the three divided clusters analyzed by IPA are described. *Decreased cytokines in schizophrenia. **Increased cytokines in schizophrenia.

**Figure 3:**
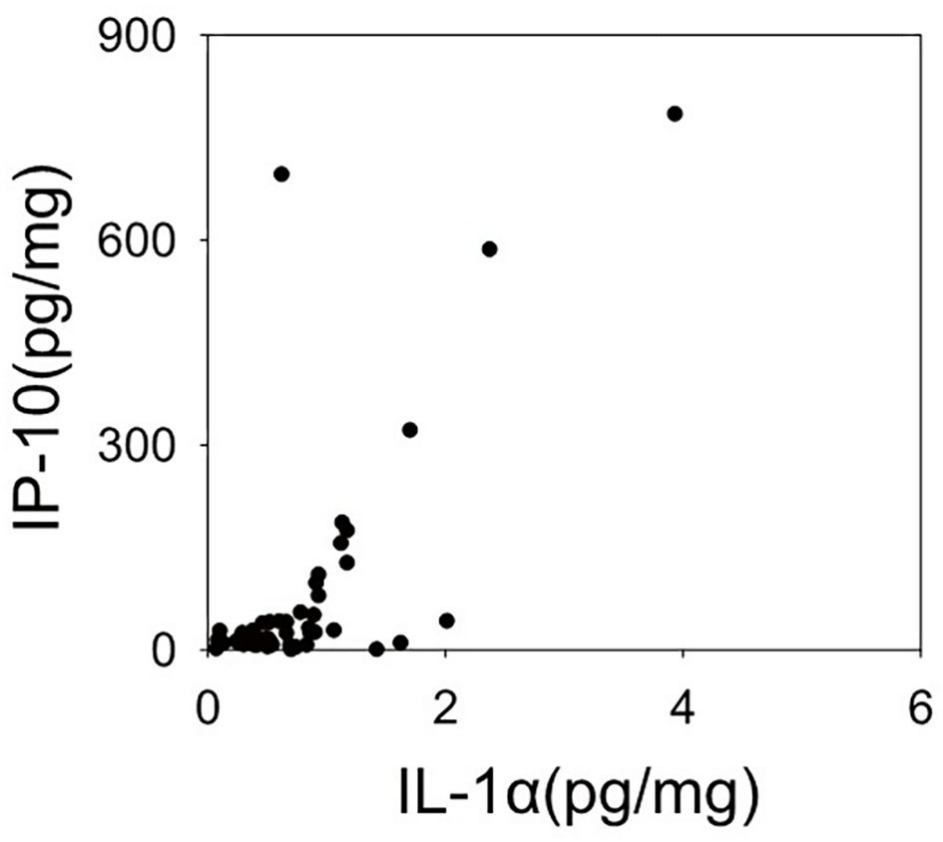
Scatter plot representing expression levels of Interleukin (IL-1α), Interferon-gamma-inducible protein 10 (IP-10). The expression levels of the two cytokines have a linear relationship. This relationship shows a significant positive correlation in a Spearman's correlation analysis (*r* = 0.583, *p* < 0.001).

Figure 2 shows the dendrogram of the expression patterns of the 20 inflammatory mediators by hierarchical cluster analysis. This dendrogram showed that the expression patterns of IL-1α and IP-10, which were significantly reduced in the schizophrenia group in this study were similar. There was a positive correlation between expression of IL-1α and IP-10 (*r* = 0.532, *P* < 0.01) (Figure 3). When classified into three clusters, IL-1α and IP-10 were classified into the same cluster 1, and IFN-α was classified into cluster 2.

In order to evaluate the validity of clustering and to connect with neuroinflammation signaling cascades, an upstream transcription factor analysis was performed. The common upstream transcription factors of inflammatory mediators belonging to each cluster obtained by analysis using IPA are shown in Figure 2. Among the transcription factors upstream of cluster 1, the top 10 most closely related by IPA analysis were BCL6, RELA, IRF9, IFI16, HMGB1, IRF5, NCOA2, ZBTB16, IRF3, and NFATC2. Similarly, the top 10 in Cluster 2 were NFKB1, STAT3, NFKBIA, NFKBIZ, NFAT5, GLI2, ZBTB7B, KLF11, ANKRD42, and IRF4. Also, the top 10 in Cluster 3 were IRF1, CITED2, IRF3, TSC22D3, TSC22D1, IRF6, NFKBIB, FOSL1, IFI16, and KLF6. The top 10 transcription factors corresponding to each cluster did not overlap between clusters, pointing to specific interactions between transcription factors and inflammatory mediators within the clusters.

## Discussion

In this study, we investigated protein expression of 30 inflammatory mediators in the STG of patients with schizophrenia by multiplex assay. This current study is the first to report a comprehensively investigated protein expression of inflammatory mediators in the STG. Of the 20 inflammatory mediators whose expression could be measured, there was a decrease in the expression of IL-1α and IP-10, and an increase in the expression of IFN-α. However, the inflammatory mediators that showed alterations (IL-1α, IP-10, and IFN-α) also did not remain significant after multiple testing. This may be due to the relatively small sample size used in this study, and the heterogeneity in the involvement of neuroinflammation in the pathology of schizophrenia (18).

A previously published mRNA expression study by Schmitt et al. (19) reported decreased gene expression of IL-1α in the STG of patients with schizophrenia. However, a meta-analysis reported no alterations in the gene expression of IL-1α and IP-10 in patients with schizophrenia (12, 13, 20). Thus, the expression of IL-1α and IP-10 in the brain may be suppressed in the chronic phase of schizophrenia observed in postmortem brain. To determine why these two inflammatory mediators are suppressed, we performed a cluster analysis of upstream transcription factors that might be involved in regulation of cytokine expression. Hierarchical cluster analysis of expression patterns of the 20 inflammatory mediators examined in this study classified IL-1α and IP-10 to the closest positions. The expression of these two inflammatory mediators also showed a linear relationship in the scatter plot. Moreover, in this upstream analysis using IPA, the top 10 transcription factors were listed as common upstream transcription factors of cluster 1, including IL-1α and IP-10, and many of these 10 transcription factors were different from the upstream transcription factors of the other two clusters. This suggests that in the chronic phase of schizophrenia, mRNA expressions of inflammatory mediators belonging to cluster 1 may be suppressed by the regulation of upstream transcription factors such as BCL6, RELA, and IRF9.

RELA, which was a common upstream factor of cluster 1 in this study, was reported to be associated with the pathology of schizophrenia. The *RELA* gene is located on chromosome 11q13, which is suggested to be linked to schizophrenia. *RELA* gene expression was also known to be downregulated in the postmortem tissue of STG in schizophrenia, as observed in its protein and gene expression levels (21). Furthermore, several SNP variants of RELA were associated with deficits in pre-pulse inhibition, which is a typical intermediate phenotype of schizophrenia (22). Taken together, the contribution of RELA to the etiology of schizophrenia might be mediated by the alternation of the expression of inflammatory mediators.

The expression of IFN-α was increased in patients with schizophrenia. To the best of our knowledge, there have been no postmortem brain studies that have reported alterations in IFN-α expression in schizophrenia. Several reports have revealed no significant differences between schizophrenia and controls in terms of plasma IFN-α levels (23, 24). However, from a clinical point of view, it has been known that IFN-α treatment is strongly associated with mental symptoms such as depression and psychosis (25, 26). Thus, elevated endogenous IFN-α levels in the chronic phase of schizophrenia may be the common biological change that causes depression.

An upstream transcription factor analysis revealed the transcription factors that specifically corresponded to each cluster of inflammatory mediators. The results of the clustering can be explained at the transcriptional regulatory level, demonstrating that the clustering in this study was biologically supported. At the same time, the process of searching for transcription factors from clusters and the revealed transcription factors in this study may lend support to the interpretation of the results of transcriptional regulatory analyses, such as a chromatin immunoprecipitation (ChIP) analysis, DNA methylation analysis, and SNP analysis. It should however be noted that the upstream transcription factors obtained by IPA analysis were based on systemic inflammation and not neuroinflammation, since the entire pathway of neuroinflammation has not yet been elucidated. In order to reinforce these findings, further research needs to be performed.

In the interpretation of these results, the effect of confounders should be considered. In this study, sex, age, and pH did not differ between schizophrenia and controls; hence, they were not considered. Previous studies have reported that antipsychotics reduce IL-1α and IP-10 expression in plasma inflammatory mediators (27–29) and dendritic cells in culture (30). However, low expressions of IL-1α and IP-10 in schizophrenia may not be due to antipsychotic medication since in the current study, there was no significant correlation between CPZeq and IL-1α and 1P-10 expression.

## Limitations

This study had some limitations. Since the study was conducted on postmortem tissue, the effects of inflammation from the underlying cause of death cannot be excluded. In addition, our study population was relatively small, limiting methods for grouping cytokine expression patterns. Therefore, we needed to classify the expression patterns in all cases, including schizophrenia and control groups. Our findings must be confirmed via postmortem examination in a larger brain cohort.

Some of the inflammatory mediators that could not be quantified might show their low expressions in the STG. According to the data of the HPA RNA-sequencing normal tissues project (31), mRNA expression of the same inflammatory mediators as above seem to be also expressed at very low levels in the cerebral cortex, such as eotaxin, IFN-γ, IL-17, IL-2, IL-3, IL-4, IL-5, and MDC (CCL22). However, two recent papers that performed a multiplex protein analysis of inflammatory mediators with brain tissue were able to detect IL2 (32) and IFN-γ (33). There may have been a certain degree of uncertainty of affinity between the panel and brain tissue lysate in this study, as no previous studies have yet performed measurements using this panel in postmortem brain tissue. To confirm the validity of this measurement, we performed a correlation analysis between the protein and mRNA expression levels using preliminary incomplete mRNA expression data for only schizophrenia cases. A significant positive correlation was found in five of 20 molecules after performing Spearman's rank correlation test (GCSF: *r* = 0.62, *p* < 0.01; IL-15: *r* = 0.55, *p* = 0.01; IL-1β: *r* = 0.37, *p* = 0.03; IL-6: *r* = 0.51, *p* = 0.02; MCP: *r* = 0.77, *p* <0.01). It is known that changes in the mRNA levels do not always reflect those of protein levels (34); therefore, we considered that this measurement was valid. However, further research is warranted to compare the mRNA expression level of inflammatory mediators measured in this study among case–controls.

## Conclusion

Based on this study, the largely unchanged major inflammatory mediators expressions in the STG of patients with schizophrenia in the chronic phase is supported by previous studies involving PFC and DLPFC brain regions. The decreased expression levels of IL-1α and IP-10 and the strong correlation between the expression of these two inflammatory mediators suggest a suppressed common upstream pathway. During the chronic phase of schizophrenia, IFN-α might be elevated. These changes may represent post-sensitization changes of inflammatory mediators in the chronic phase of schizophrenia caused by changes in inflammatory cytokines such as IL-1β, TNF-α, and INF-γ in the acute phase. However, it should be noted that these alterations did not remain significant after multiple testing. These results provide insight into post-inflammatory alternation in chronic schizophrenia. As research on neuroinflammation progresses, further research is needed to better understand the relationship between schizophrenia and neuroinflammation.

## Data Availability Statement

The original contributions presented in the study are included in the article/supplementary material, further inquiries can be directed to the corresponding author/s.

## Ethics Statement

The studies involving human participants were reviewed and approved by Fukushima Medical University. The patients/participants provided their written informed consent to participate in this study.

## Author Contributions

RI, MH, AW, YK, and KK designed the study. RI, MH, and AW performed the experiments. RI, YK, MH, AW, AN, AK, MS, and HY collected postmortem brain samples and clinical information. RI, MH, TK, and TM undertook the statistical analysis. RI wrote the first draft. All authors contributed to and have approved the final manuscript.

## Conflict of Interest

The authors declare that the research was conducted in the absence of any commercial or financial relationships that could be construed as a potential conflict of interest.

## Abbreviations

STG: Superior temporal gyrus
CPZeq: Chlorpromazine equivalents
ELISA: Enzyme-linked immunosorbent assay
IL: Interleukin
GM-CSF: Granulocyte macrophage colony-stimulating factor
IFN: Interferon
MCP: Monocyte chemotactic protein
TNF: Tumor necrosis factor
G-CSF: Granulocyte colony stimulating factor
IP: Interferon-gamma-inducible protein
MIP: macrophage inflammatory protein
EGF: epidermal growth factor
VEGF: Vascular endothelial growth factor
MDC: macrophage-derived chemokine.
